# Subcritical Water-Carbon Dioxide Pretreatment of Oil Palm Mesocarp Fiber for Xylooligosaccharide and Glucose Production

**DOI:** 10.3390/molecules23061310

**Published:** 2018-05-30

**Authors:** Norlailiza Ahmad, Mohd Rafein Zakaria, Mohd Zulkhairi Mohd Yusoff, Shinji Fujimoto, Hiroyuki Inoue, Hidayah Ariffin, Mohd Ali Hassan, Yoshihoto Shirai

**Affiliations:** 1 Department of Bioprocess Technology, Faculty of Biotechnology and Biomolecular Sciences, Universiti Putra Malaysia, Serdang, Selangor 43400 UPM, Malaysia; lailizaahmad@gmail.com (N.A.); mzulkhairi@upm.edu.my (M.Z.M.Y.); hidayah@upm.edu.my (H.A.); alihas@upm.edu.my (M.A.H.); 2 Laboratory of Biopolymer and Derivatives, Institute of Tropical Forestry and Forest Products, Universiti Putra Malaysia, Serdang, Selangor 43400 UPM, Malaysia; 3 Research Institute for Sustainable Chemistry, National Institute of Advanced Industrial Science and Technology (AIST), 3-11-32 Kagamiyama, Higashi-Hiroshima, Hiroshima 739-0046, Japan; s.fujimoto@aist.go.jp (S.F.); inoue-h@aist.go.jp (H.I.); 4 Department of Biological Functions and Engineering, Graduate School of Life Science and Systems Engineering, Kyushu Institute of Technology, Fukuoka 804-8550, Japan; shirai@life.kyutech.ac.jp

**Keywords:** oil palm mesocarp fiber, subcritical H_2_O-CO_2_, pretreatment, xylooligosaccharides, glucose

## Abstract

The present work aimed to investigate the pretreatment of oil palm mesocarp fiber (OPMF) in subcritical H_2_O-CO_2_ at a temperature range from 150–200 °C and 20–180 min with CO_2_ pressure from 3–5 MPa. The pretreated solids and liquids from this process were separated by filtration and characterized. Xylooligosaccharides (XOs), sugar monomers, acids, furans and phenols in the pretreated liquids were analyzed by using HPLC. XOs with a degree of polymerization X2–X4 comprising xylobiose, xylotriose, xylotetraose were analyzed by using HPAEC-PAD. Enzymatic hydrolysis was performed on cellulose-rich pretreated solids to observe xylose and glucose production. An optimal condition for XOs production was achieved at 180 °C, 60 min, 3 MPa and the highest XOs obtained was 81.60 mg/g which corresponded to 36.59% of XOs yield from total xylan of OPMF. The highest xylose and glucose yields obtained from pretreated solids were 29.96% and 84.65%, respectively at cellulase loading of 10 FPU/g-substrate.

## 1. Introduction

Malaysia is the second largest oil palm producer, with more than 15 million tonnes of palm oil produced, along with the production of biomass such as oil palm empty fruit bunch (OPEFB), oil palm mesocarp fiber (OPMF) and oil palm frond fiber (OPFF) [1]. OPMF is one of the potential and attractive biomass which can be used as a biomaterial to produce many bio-products such as biosugar, biogas, biochar and biocomposite which can be further used by various industries. Generally, OPMF consists of cellulose (23–29%), hemicellulose (21–34%), lignin (21–32%), extractives and ash [2,3,4]. Due to the complex structure of OPMF, different pretreatments have been performed to disrupt the lignocellulose structure to give maximum access of enzymes to hemicellulose and cellulose [2,5]. Autohydrolysis is one of the preferable pretreatment methods as it uses a green approach such as compressed hot water with various reaction temperatures and times to hydrolyze xylan into shorter oligosaccharides such as xylooligosaccharides (XOs) and xylose [6,7]. Recently, subcritical H_2_O-CO_2_ pretreatment has become more attractive as it offers benefits such as the mild conditions used, less formation of undesirable by-products and the use of non-toxic gases [8]. Subcritical H_2_O-CO_2_ pretreatment produces carbonic acid that facilitates the hydrolysis of hemicellulose in biomass with no negative impact on the environment since when the pressure is released, the gas will be neutralized [9,10]. It was reported that high-pressure CO_2_ penetrates the small pores in the biomass and helps in disrupting the biomass structure, thus improving the hydrolysis rate of hemicellulose in the biomass [8,11]. The combined severity factor (CS_PCO2_) is used to evaluate the influence of temperature, time and high-pressure CO_2_ on the hydrolysis of xylan [8,9,10,11,12].

The structure, degree of polymerization (DP) and yield of XOs depend on the type of biomass and methods used in the production stage [12]. XOs can be obtained abundantly in pretreatment liquids, together with undesired by-products such as acetic acid, furfural, 5-hydroxymethylfurfural (5-HMF) and tannic acid [11,13,14]. Purification steps are necessary to remove these undesired by-products to obtain high purity XOs. Different types of XOs can be produced from xylan such as xylobiose (X2), xylotriose (X3), xylotetraose (X4) and xylopentaose (X5) [15,16]. XOs are produced from corncobs [6], cotton stalks, tobacco stalks, sunflower stalks, wheat straw [17], sugarcane bagasse [13], OPFF [18] and OPEFB [16].

Oil palm biomass such as OPEFB and OPFF were reported to produce XOs by an autohydrolysis process [16,18]. Under optimal pretreatment conditions, 17.6 g/L of XOs was produced from OPEFB at pretreatment severity log *R*o = 3.91 (210 °C) with DP X5–X40 and 6.15 g/L with DP X5–X10 was recorded [16]. Autohydrolysis pretreatment of OPFF at 121 °C for 60 min and subsequent enzymatic hydrolysis with xylanase at 8 U/100 mg of autohydrolyzate resulted in the production of 17.5% and 13.9% of XOs and xylobiose, respectively [16]. It was reported that in hydrothermal pretreatment of OPMF monomeric xylose and XOs were produced at pretreatment severity log, *R*o = 3.25–3.94, and XOs concentrations were detected in the range from 5.0 to 7.0 g/L. It was suggested that OPMF is a suitable biomass to produce XOs [19]. It is worth noting that in all previously reported oil palm biomass experiments autohydrolysis processes alone were conducted and the formation of XOs was obtained at high pretreatment severities and with subsequent enzymatic hydrolysis by xylanase. Addition of CO_2_ in the autohydrolysis process and operation under subcritical and supercritical conditions offered several advantages over autohydrolysis alone [7,8,9]. An attempt to obtain a higher XO yield from OPMF under mild operational conditions with the application of initial pressurized CO_2_ (0–5 MPa) was performed in this study. 

Due to the high demand and potential uses of XOs in the industry as well as abundant sources of OPMF from the oil palm industry, the present study aimed to evaluate the production of XOs and glucose from OPMF using subcritical H_2_O-CO_2_ pretreatment under isothermal and non-isothermal conditions. The present work was also conducted to prove that impregnation of CO_2_ in the subcritical H_2_O reaction could reduce the formation of inhibitory by-products resulting in the improved production of XOs compared to subcritical H_2_O without CO_2_ assistance. The efficiency of the pretreatment was evaluated based on the types and concentration of XOS produced and glucose yield from cellulose conversion by enzymatic hydrolysis. To the best of our knowledge, this is the first study on OPMF for XOs and glucose production under subcritical H_2_O-CO_2_ pretreatment process conditions.

## 2. Results and Discussion

### 2.1. Compositional Analysis

The chemical compositions of the biomass were complex and varied according to its structure and origin. Table 1 shows that OPMF used in this study mainly comprised cellulose (23.6%), hemicellulose (22.3%), lignin (28.2%), and solvent extractives (8.3%) and was within the range of earlier reports [2,3]. Different pretreatments were performed first to find the best pretreatment of OPMF as it contains a high lignin content compared to other oil palm biomass [4,19]. The determined hemicellulose content was relatively similar to that of other lignocellulosic materials such as tobacco stalks, wheat straws, corn stover and olive stones, with contents of 20.0%, 20.9%, 22.0% and 23.3%, respectively [17,20,21]. All of these biomasses were potentially used as substrates to produce XOs. The present finding showed that OPMF was among the biomasses that could potentially be used as raw materials for XOs production.

### 2.2. Physico-Chemical Properties of Untreated and Pretreated Samples

#### 2.2.1. Solids Recovery

Subcritical H_2_O-CO_2_ pretreatment is one of the thermochemical methods that aim to disrupt the hemicellulose structure and break down xylan into a smaller chain of XOs and xylose. In this study, CS_PCO2_ was used to evaluate the effect of temperature, time and pressure of CO_2_ on the hydrothermal process of OPMF. The CS_PCO2_ was used to monitor hydrothermal reaction by pH value obtained from Henry’s law equation and to facilitate the efficiency of pretreatment [9]. As shown in Table 2, subcritical H_2_O-CO_2_ were performed at 150–200 °C for 20–60 min at 0, 3, 5 MPa which corresponds to CS_PCO2_ = −0.93 to −0.06 and the physicochemical properties were compared with subcritical H_2_O. It was observed that pretreated solids recovery was in the range from 62.6–84.1% and decreased towards increasing CS_PCO2_ and reached 62.6% at the final CS_PCO2_= −0.06. The reduction of solid recovery yields towards higher CS_PCO2_ can be explained from xylan solubilization into pretreated liquids [6]. 

#### 2.2.2. Xylooligosaccharide Content in the Pretreatment Liquids

In pretreatment liquids, hemicellulose was observed to depolymerize into xylan-derived products such as xylose, XOs, arabinose and furfural during hydrothermal pretreatment under the conditions tested (Table 2). XOs represented the major compound present in the pretreatment conditions examined and the increased of XOs production were observed from CS_PCO2_= −0.93 to −0.19 due to the higher solubilization of hemicellulose components concomitant with higher pretreatment severities. As pretreatment severity increased, the XOs yield started to decrease at CS_PCO2_ = −0.06 and this corresponded to a sharp increase of xylose monomer concentration up to 16.40 mg/g at CS_PCO2_ = −0.06. The highest XOs was recorded at CS_PCO2_ = −0.19 (180 °C, 60 min, 3 MPa), with 8.16 g/L and this value was equivalent to 36.6% of XOs yield from xylan and corresponded to 81.60 mg/g of raw OPMF. At this condition, xylose and furfural were recorded with concentrations of 1.85 g/L and 14.13 g/L, respectively. 

Approximately 49.15% of the total xylan was degraded to major compound XOs, followed by xylose and furfural. The XOs concentration obtained in this study was slightly lower compared to that reported by Morais et al. [8] using a wheat straw with XOs production of 11.4 g/L which corresponded to 61.7% of XOs from the total xylan at CS_PCO2_ = −0.33 (215 °C, 30 bar CO_2_). Ho et al. [16] found the highest XOs concentration with 17.6 g/L was obtained from OPEFB by autohydrolysis pretreatment at log, *R*o = 3.91. Therefore XOs was estimated based on xylose and arabinose and 6.15 g/L of XOs with DP X5–X10 was obtained. Interestingly, in this study, by comparing XOs yield from subcritical H_2_O-CO_2_ at CS_PCO2_ = −0.93 with subcritical H_2_O treatment, the XOs value was increased from 1.12 g/L to 1.66 g/L which corresponded to a 48.2% increment. In another study, Zakaria et al. [19] reported XOs were found to be a major compound with the highest value, 7.0 g/L at severity factor log, *R*o = 3.94 from OPMF after the hydrothermal process. 

The present study has shown that the impregnation of CO_2_ in the hydrothermal process has improved XOs production. Sabiha-Hanim et al. [18] reported a maximum of 48% of the hemicellulose was hydrolyzed using an autoclave system (121 °C, 20–80 min). In term of competitive XOs yields from different biomass, Otieno and Ahring [22] has performed autohydrolysis pretreatment at 145 °C for 60 min on *Miscanthus sinensis*, *Panicum virgatum*, *Calamagroustis acutiflora* and bagasse and found that XOs yields were 65.0%, 84.2%, 87.9% and 92.3%, respectively even though the initial dry mass of xylan was >20%. Lower xylan conversion to XOs probably due to aggregation of xylan with lignin during repolymerization that formed precipitates upon cooling process [23]. It can be concluded that the production of XOs heavily dependent on the types of biomass and selection of pretreatment conditions tested such as temperature, reaction time, initial CO_2_ pressure and solid to liquid ratio [13,14]. 

#### 2.2.3. Monomeric Sugars, Acids, Furans and Tannic Acids Content in the Pretreatment Liquids

Other monomeric sugars like glucose and arabinose were detected in low concentrations in the pretreated liquids. Glucose amount at all conditions was recorded low from 0.12 g/L to 0.30 g/L indicated that this treatment only caused small solubilization of cellulose into the pretreated liquids [14]. Garrote et al. [6] reported a maximum value of glucose in the pretreated liquid of only 0.8 g/L which indicated that hydrothermal treatment at 160–220 °C did not affect the cellulose structure of the biomass. Other by-products produced from hydrothermal process heavily depending on the types of materials and the pretreatment conditions applied. The acidic condition created during hydrothermal process released by-products such as acetic acid, 5-HMF, furfural, formic acid and tannic acid [24]. As shown in Table 2 acetic acid concentration increases as the CS_PCO2_ increases and achieved a maximum value at CS_PCO2_ = −0.06 with 381.60 mg/g of raw OPMF. The increasing trend of acetic acid showed that the breakdown of hemicellulose components and xylan side-chains occurred in this pretreatment process and acetic acid can act as a catalyst in carbohydrate degradation [6,14]. 

Furfural and 5-HMF were formed from degradation of pentose and hexose sugars, respectively and further degradation of furfural and 5-HMF produced formic acid [7,21,24]. The trend of XOs, xylose and furfural over CS_PCO2_ showed a correlation of degradation of xylan-derived product from OPMF. As the severity increased, the XOs concentrations decreased and xylose concentration increased which indicated the sugar degradation occurred caused by the severe pretreatment conditions [14]. Tannic acids were soluble degradation by-product from lignin component formed during the hydrothermal process. This finding was in agreement with a previous study by Zakaria et al. [19], whereby tannic acid was affected by the treatment severities. 

#### 2.2.4. pH of the Pretreatment Liquids

The pH of pretreatment liquids presented in Table 2 was calculated using the van Walsum equation [9] and measured pHs were recorded in the range from 4.16–4.32 across all conditions tested. It was obvious that pH of the pretreated liquids in subcritical H_2_O-CO_2_ was more acidic in comparison to subcritical H_2_O case, probably due to the presence of carbonic acid formed from the reaction of H_2_O and CO_2_ in the reactor during pretreatment process [9] together with higher concentrations of acetic acid and formic acids. Lower pH values obtained from subcritical H_2_O pretreatment at higher pretreatment severity were probably due to acetic acid accumulation caused by cleavage of acetyl groups during the hemicellulose degradation [25]. On the other hand, the presence of high dense CO_2_ and hot water promoted gas diffusion into the biomass and caused more hemicellulose disruption [26].

### 2.3. Types of XOs Produced

**Figure 1:**
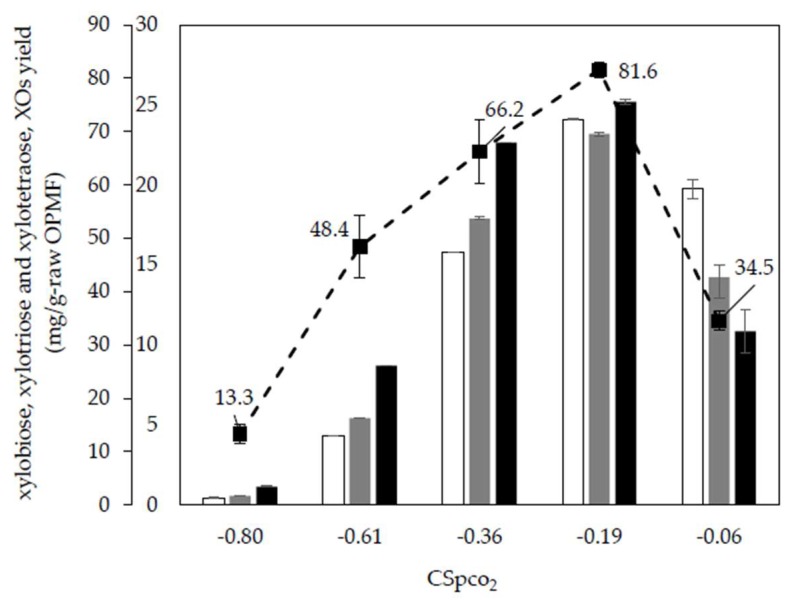
XOs characterization (with respective error bar) for (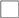) xylobiose, (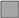) xylotriose, (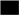) xylotetraose, (- - -) XOs yield.

Figure 1 shows a yield of XOs over combined severity factor, CS_PCO2_ and the characteristics of XOs from pretreatment liquid samples were determined based on their degree of polymerization by using Dionex ICS 3000. Xylobiose (DP X2), xylotriose (DP X3) and xylotetraose (DP X4) were XOs obtained from pretreatment liquid samples and the highest XOs yield was obtained at CS_PCO2_= −0.19 (180 °C, 60 min, 3 MPa) with 8.16 g/L. This value was equivalent to DP X2–X4 of the total xylan and 81.60 mg/g of raw OPMF. Under these conditions, xylobiose, xylotriose and xylotetraose were recorded with concentrations of 24.11 mg/g, 23.18 mg/g and 25.19 mg/g, respectively. From this value, 88.82% of total XOs obtained have DP X2–X4 and the rest of XOs was probably in higher DP form. Higher concentration of XOs yields for DP X2–X4 was recorded at higher pretreatment severity and this can be explained that at a higher temperature and longer reaction time, most of the longer chain XOs were degraded to shorter chain length oligosaccharides and other by-products such as furfural, therefore lower amount of XOs was recovered [28]. 

Sabiha-Hanim et al. [18] reported that XOs from OPFF after hydrothermal treatment at 121 °C for 60 min contain mainly xylobiose and xylotriose and after subjection to enzymatic hydrolysis using xylanase from *Trichoderma viride*. The XOs that mainly comprise DP X2–X6 were also observed in other agricultural wastes such as tobacco stalks, cotton stalks, sunflower stalks and wheat straw from acid hydrolysis processed [17]. In other study using different oil palm biomass, Ho and co-workers [16] found that XOs obtained from empty fruit bunch (EFB) was mainly with DP X5–X40 with XOs concentration 17.64 g/L after underwent autohydrolysis process at log *R*o = 3.91. Similarly, XOs generated from xylan of natural grass using enzymatic hydrolysis with *Trichoderma viride* was recorded containing major xylobiose (11.0%) and a small amount of xylotriose [29]. 

It is worth noting that most of the previous studies on types of XOs detected from xylan involved an additional biological treatment such as enzymatic hydrolysis and acid hydrolysis. In contrast, the type of XOs detected in the present work were only from the subcritical H_2_O-CO_2_ pretreatment process without further treatment by any other hydrolysis process. Hence, the type and DP of XOs exhibited mainly depend on the hydrolysis treatment and condition used [13]. XOs have novel applications in many industries such as the food, pharmaceutical and health industries. The XOs with short DP range from DP X1–X6 have a beneficial and advantageous function as prebiotics in food-related products [30]. XOs are potential compounds that can behave as prebiotics when ingested as it can stimulate beneficial bacteria inside the colon [31]. 

Xylobiose (DP X2) has been found to be an important oligosaccharide in the food industry and was reported to have 30% sweetness of sucrose, while other XOs exhibited less sweetness. This has resulted in xylobiose as the main target in food-related products. Besides that, the use of XOs as a food ingredient can help to produce specific food to promote health and reduce the risk of side effect.

### 2.4. Enzymatic Hydrolysis of Pretreated Solids

Essentially, most of the cellulose component remained in the pretreated solids and only a small portion was solubilized in the pretreatment liquid [6,21]. Thus, instead of high XOs obtained from the pretreatment liquid samples, the conversion of sugars from solid samples was also studied. Table 3 summarizes chemical compositions and physical properties of untreated, subcritical water and subcritical water-CO_2_ pretreatments of OPMF. It was observed that cellulose contents were increased towards higher pretreatment severities and recorded the highest cellulose content, 36.67% at CS_PCO2_ = 0.06 (190 °C, 60 min, 3 MPa). In contrast, hemicellulose content was decreased when higher pretreatment severities were applied. The lowest hemicellulose content obtained was 3.14% at CS_PCO2_ = 0.06, indicating successful removal of hemicellulose. 

Sugar yields from xylose and glucose obtained from enzymatic hydrolysis of selected pretreated solid samples are presented in this section. Xylose and glucose yields were compared with untreated, subcritical H_2_O and subcritical H_2_O-CO_2_ pretreatments. Xylose and glucose yields from untreated OPMF recorded the lowest compared to all pretreated samples. Approximately 4% increment of glucose yield was obtained when OPMF was hydrothermally pretreated at 150 °C, for 60 min. The enzymatic hydrolysis of the untreated sample yielded lower glucose concentration since *Acremonium cellulase* has less accessibility to cellulose when high hemicellulose present in the biomass. This finding was in agreement with the study by Hsu et al. [32], where higher sugar yield can be achieved at higher hemicellulose removal which provided more accessibility of cellulase to cellulose. Zakaria et al. [4] reported obvious hemicellulose dissolution of OPEFB and OPFF was obtained when using hot compressed water (HCW) treatment at condition temperature ranges from 170–190 °C for 20 min and 10 min, respectively that resulted in high conversion yield of cellulose to glucose.

### 2.5. Cellulose Crystallinity Index 

From Table 3, the Crl values of untreated OPMF were lower (52.35%) compared to subcritical H_2_O treatment (62.35%). The CrI values for pretreated solids under subcritical H_2_O-CO_2_ pretreatment were increased as the pretreatment intensity increased. This can be explained by the removal of amorphous hemicellulose from the solid samples towards higher pretreatment condition. Besides the presence of hemicellulose, enzymatic attack on cellulose can also be influenced by cellulose crystallinity of the biomass [8]. The increase Crl of cellulose represented the disclosure amount of cellulose that susceptible to the enzymatic attack in the sample [5]. 

### 2.6. Specific Surface Area

Hsu et al. [32] found that SSA and PV of pretreated solid structure affected the enzymatic hydrolysis of biomass. It was obvious that subcritical H_2_O-CO_2_ improved xylose and glucose yields and this might due to higher SSA and PV obtained after removal of xylan from the cellulose-hemicellulose-lignin matrix. Therefore, inconsistent values of SSA and PV in the pretreated solids are probably due to errors caused by redeposition of lignin or pseudolignin onto the surface of pretreated solids [33]. From Table 3, glucose yield from pretreated solid samples increased in line with increasing CS_PCO2_, indicating the higher efficiency of the enzymatic hydrolysis. Xylose yields were also increased in line with increasing CS_PCO2_ and started to decrease at CS_PCO2_ = 0.06 due to the lower xylan content in the pretreated solids. Morphological characteristics of pretreated solid samples were analyzed using SEM to observe the effect of subcritical H_2_O-CO_2_ pretreatment on the surface of pretreated solid samples. The glucose yields obtained from enzymatic hydrolysis of pretreated solids were compared with a previous work [19]. At the same pretreatment severities log, *R*o = 3.66 and log, *R*o = 3.73, an addition of initial pressure of CO_2_ at 3 and 5 MPa have resulted in an increment of glucose yields by 12.9% and 26.5%, respectively (Table 4). This finding has proven the autohydrolysis process with CO_2_ assisted improved accessibility of cellulase to cellulose, which is economically feasible at a commercial scale of production. 

### 2.7. SEM Analysis

**Figure 2:**
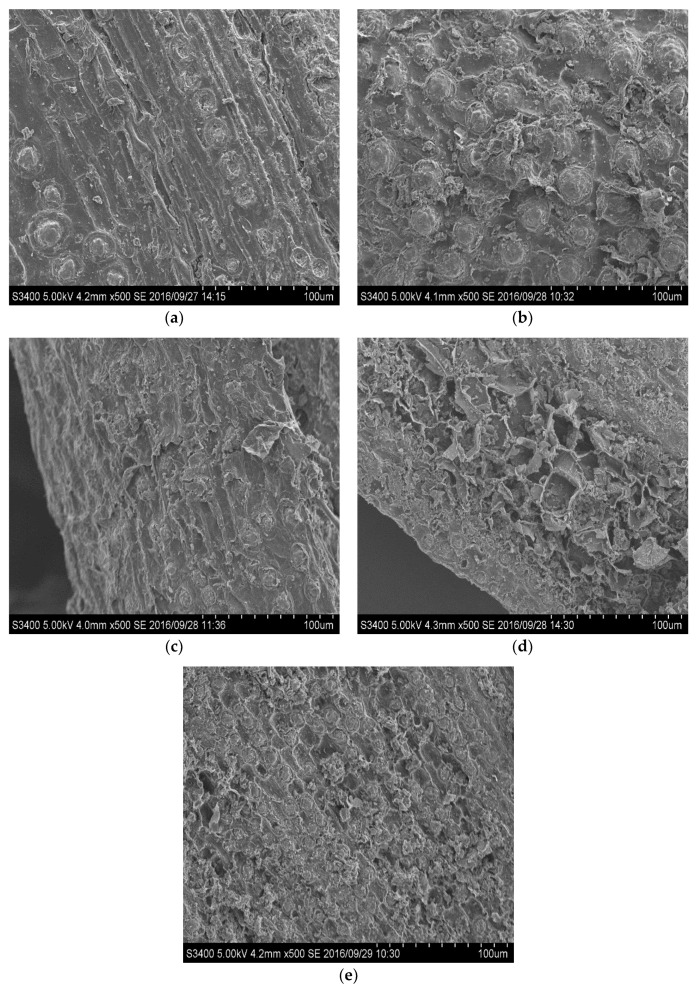
SEM micrographs of (**a**) untreated OPMF (**b**) subcritical H_2_O treatment at 150 °C, 60 min (**c**) subcritical H_2_O-CO_2_ treatment at 150 °C, 60 min 5 MPa (**d**) subcritical H_2_O-CO_2_ treatment at 170 °C, 40 min, 3 MPa (**e**) subcritical H_2_O-CO_2_ treatment at 190 °C, 60 min, 3 MPa obtained with magnification 500×.

Morphological characteristics of pretreated solid samples were analyzed using SEM to observe the effect of subcritical H_2_O-CO_2_ pretreatment on the surface of pretreated solid samples Figure 2. After hydrothermal treatment, physical changes can be noticed on the surface of the pretreated solids compared to untreated solid. The untreated OPMF (Figure 2a) showed a rigid and intact surface of biomass, which provides less accessibility of enzyme penetration into cellulose component. Meanwhile, for all pretreated solids (Figure 2b–e), rough surface and more porous fibers were observed. When comparing to subcritical H_2_O pretreatment, impregnation of CO_2_ was more likely have a rougher surface and the peeling-off of the outer layer of the cell wall compared to the sample without CO_2_ effect (Figure 2b,c) and the surface changes at the more severe condition in CO_2_ reaction (Figure 2d,e). The blending effect of H_2_O and CO_2_ help to disrupt and fractionated more fiber, increased SSA and PV and made cellulose accessible to enzymatic attack [26,34]. 

## 3. Materials and Methods

### 3.1. Raw Material Preparation

OPMF used in this study was collected from Seri Ulu Langat Palm Oil Mill, Dengkil, Selangor, Malaysia. The samples were sun-dried for two days and crushed kernels and shells were manually separated from OPMF fibers before compositional analysis and other experimental work to avoid error in data analysis. The samples were ground to 2 mm size using a Pulverisette 15 cutting mill (Fritsch, Idar-Oberstein, Germany) and dried overnight in *vacuo* at 40 °C. The samples were stored in a vacuum chamber at room temperature (24 °C) before further analysis.

### 3.2. Chemical Compositional Analysis

Chemical compositional analysis such as extractives, cellulose, hemicellulose, Klason lignin, ash and moisture content of untreated and pretreated OPMF were determined according to the method reported by Sluiter et al. [27]. 

### 3.3. Subcritical H_2_O and Subcritical H_2_O-CO_2_ Pretreatments

Subcritical H_2_O and subcritical H_2_O-CO_2_ pretreatments of OPMF were conducted in a 35 mL stainless steel tube reactor. In this study, 2 mm size OPMF was used with solid to liquid (S: L) ratio of 1:10. 3 g of oven dried OPMF samples and 30 mL of distilled water were filled inside the reactor. The reactor was tightened closely and purged with CO_2_ at a pressure range from 0–5 MPa. The detection of gas leakage was performed to ensure there was no CO_2_ leak from the reactor. The reactor was immersed in the sand bath, with controlled temperature (150–200 °C) using automatic temperature controller. The mixture was homogenized by agitation at 60 rpm at time range from 20–240 min. After completion of the heating process, the reactor was cooled down in the water reservoir. The pressure was released at the end of the pretreatment. The solid and liquid samples were separated using filter paper No. 2 with pore size 0.5 µm (Advantec, Tokyo, Japan) and pH of the pretreatment liquids was recorded using a digital pH meter (B-712 LAQUAtwin, Horiba, Kyoto, Japan). The pretreated solids were dried at 40 °C for 48 h in a vacuum drier. The pretreatment liquids were further filtered with 0.22 µm PTFE syringe filter (Whatman, Clifton, NJ, USA) and directly injected into the HPLC for sugars and by-products determination.

### 3.4. Combined Severity Factor 

The intensity of hydrothermal process was expressed in terms of severity factor (log, *R*o) that combined the reaction temperature and time factor according to Equation (1):

(1) $$Ro\;=\;\left(t\;\exp\left[\frac{T-100}{14.75}\right]\right)$$

where *t* is time expressed in minutes, *T* is temperature expressed in °C and 14.75 is an empirical parameter related to temperature and activation energy [35]. Combined severity factor (CSF) include the pH effect on the pretreatment severity due to the presence of carbonic acid formed from the CO_2_ pressure is according to Equation (2):

(2) CSF = log Ro−pH

because it was difficult to measure the pH during the reaction to show the influence of carbonic acid, the pH values were calculated from Equation (3):

pH = 8.00 × 10^−6^ × (*T*^2^ + 0.00209) × (*T* − 0.126) × ln (P_CO2_) + 3.92 (3)

Thus, in this experiment, the combined severity factor was calculated as in Equation (4) below as recommended by van Walsum [9]:

(4) $$\mathrm{CSF}=\log(t\;\exp\left[\frac{\left(T-100\right)}{14.75}\right])-\;8.00\;\times \;10^{-6}\;\times \;T^{2}\;+\;0.00209\;\times \;T\;-\;0.126\;\times \;\ln\;\left(P_{\mathrm{CO}2}\right)\;+\;3.92$$

### 3.5. Determination of Monomeric and Total Monomeric Sugars from Pretreatment Liquids

Monomeric and total monomeric sugars such as glucose, xylose, galactose, mannose and arabinose in pretreatment liquid samples were determined by high-performance liquid chromatography (HPLC) according to the report by Inoue et al. [36]. For total monomeric sugar analysis, 5 mL of pretreatment liquid sample was hydrolyzed in diluted 4% (*v*/*v*) H_2_SO_4_ and autoclaved at 121 °C for 1 h. The sugar produced in the liquid sample was cooled and filtered using a Dionex OnGuard^TM^ 11A cartridge filter (Thermo Scientific, Waltham, MA, USA) to neutralize the pH before HPLC analysis [27]. The organic acids present in the liquid sample such as acetic acid, furfural, 5-HMF and formic acid were detected by HPLC as reported earlier [36].

### 3.6. Determination of Tannic Acid

Tannic acid concentration in the pretreatment liquids was determined using Folin-Ciolcalteu method by UV-VIS spectrophotometer (UV mini-1240, Shimadzu, Kyoto, Japan) following the method described by Makkar [37].

### 3.7. Determination Degree of Polymerization (DP) of XOs

The DP of XOs was determined by a Dionex ICS 3000 system equipped with an AS3000 auto sampler using high-performance anion exchange chromatography with a pulse amperometric detection (HPAED-PAD) system (Thermo Scientific). The types of XOs were identified by comparing the peak areas of standard xylobiose (X2), xylotriose (X3) (Wako, Osaka, Japan) xylotetraose (X4) (Biocon, Nagoya, Japan). A Carbopac PA1 column (4 × 250 mm, Dionex, Thermo Scientific) with PA1 guard column (4 × 50 mm, Dionex) was used at a flow rate of 1.0 mL/min and the column temperature was set at 35 °C. A pulsed amperometric detector with an Au electrode operating in the integrated amperometric mode (Dionex) was used for the detection of XOs which was separated with a gradient of 10–100 mM NaOH for 15 min, followed by 0–20 mM sodium acetate gradient in 100 mM NaOH for 25 min.

### 3.8. Enzymatic Hydrolysis

Enzymatic hydrolysis was performed by using enzyme cocktail constituting 40 FPU/mL *Acremonium* cellulase (Meiji Seika Co., Tokyo, Japan) and 10% Optimash BG (Genencor International, Rochester, CA, USA). The enzymatic assays were performed in 6% substrate loading. In a standard assay, 10 FPU/g substrate of *Acremonium* cellulase and 0.1% final concentration from Optimash BG stock were added to 0.09 g of the substrate in a final concentration of 50 mM sodium acetate buffer (pH 5.0). The mixture was added up to 1.5 mL total volume. The activities of enzyme cocktail in the reaction mixture as follows: FPase, 0.33 FPU/mL; xylanase, 32.5 U/mL; carboxymethyl cellulase, 7.4 U/mL; β-glucosidase, 1.8 U/mL; β-xylosidase, 0.03 U/mL. The enzymatic hydrolysis was performed at 50 °C for 72 h with shaking. The experiment was performed in triplicate and average results were presented. The sugar yield was calculated using Equation (5):

Sugar yield (%) = [weight of monomeric sugars after enzymatic hydrolysis/weight of total monomeric sugars from the untreated sample after hydrolysis using H_2_SO_4_] × 100 (5)

### 3.9. SEM, BET and CrI Analyses

The untreated and pretreated OPMF samples were sputtered with Pt-Pd for 100 s (Ion sputterer; Hitachi, Tokyo, Japan). The coated samples were examined by field emission scanning electron microscopy (S-3400N, Hitachi, Japan) at 1 kV. The solids were rinsed with ethanol then soaked with t-butyl alcohol and dried before SEM analysis [19]. 

The specific surface area of the sample was determined from the Brunauer-Emmett-Teller (BET) plot of nitrogen adsorption-desorption isotherms [38]. The total pore volume was determined at At *P*/*P*_0_ = 0.99. 

Wide angle X-ray diffraction (WAXD) patterns analysis of the untreated and hydrothermally treated OPMF samples were determined by RINT-TTR III X-ray diffractometer (Rigaku, Tokyo, Japan) as reported earlier [15]. 

The crystallinity index (CrI) was calculated using Equation (6) based on the method of Segal et al. [39]:

*Crystallinity index* (%) = [(*I*_002_ − *I*_am_)/*I*_002_] × 100 (6)

*I*_002_: The intensity at about 2*θ* = 22.2°
*I*_am_: The intensity at 2*θ* = 17.6°

## 4. Conclusions

Subcritical H_2_O-CO_2_ pretreatment of OPMF was successfully performed under an optimal condition at CSP_CO2_= −0.19 (180 °C, 60 min, 3 MPa). Approximately 8.16 g/L of XOs was produced, which equivalent to 36.59% of XOs yield from xylan. xylobiose, xylotriose and xylotetraose with DP X2–X4 were the XOs identified in the pretreatment liquids. Enzymatic hydrolysis of OPMF pretreated solids at higher pretreatment severities showed that high amount of glucose could be produced. To the best of our knowledge, this is the first report on subcritical H_2_O-CO_2_ pretreatment of OPMF for the production of XOs and its potential in industrial applications. Purification of XOs produced from OPMF using several methodologies and future application of XOs are in research in development progress.

## Figures and Tables

**Table 1:** Chemical composition of OPMF used in this study in comparison to previous reports.

Chemical Component	Content (wt %)			
Solvent extractives	8.3 ± 0.4 ^a^	11.4 ± 0.2 ^b^	6.3 ± 0.51 ^a^	-
Cellulose	23.6 ± 0.9	25.0 ± 1.7	28.8 ± 0.48	42.8 ± 0.69
Hemicellulose	22.3 ± 0.5	25.7 ± 3.3	25.3 ± 0.65	33.1 ± 2.01
Klason Lignin	28.2 ± 1.4	25.5 ± 0.5	28.9 ± 2.07	20.5 ± 3.44
Ash	5.8 ± 0.7	5.8 ± 0.2	2.6 ± 0.34	3.6 ± 0.74
Reference	This study	Zakaria et al. [5]	Iberahim et al. [2]	Nordin et al. [3]

‘-’ Not determined; ^a^ Ethanol extractives; ^b^ Acetone extractives.

**Table 2:** Physical and chemical properties of pretreated samples under various pretreatment conditions.

Reaction Conditions	Subcritical H_2_O		Subcritical CO_2_-H_2_O													
T (°C)	150		150		160		170		170		180		180		200	
Time (min)	60		60		40		20		40		40		60		20	
Pressure (MPa)	0		3		3		5		3		3		3		5	
Log (*R*o)	3.25		3.25		3.37		3.36		3.66		3.96		4.13		4.25	
pH (pretreated liquid)	4.41		4.18		4.22		4.16		4.27		4.32		4.32		4.31	
CS_pCO2_	-		−0.93		−0.85		−0.80		−0.61		−0.36		−0.19		−0.06	
Solid recovery (*w*/*w* %)	83.29		84.14		82.41		80.37		70.22		63.25		68.45		62.56	
Composition/yields	g/L	mg/g	g/L	mg/g	g/L	mg/g	g/L	mg/g	g/L	mg/g	g/L	mg/g	g/L	mg/g	g/L	mg/g
XOs *	1.12	11.23	1.66	16.60	2.14	21.40	1.33	13.30	4.84	48.40	6.62	66.20	8.16	81.60	3.45	34.52
Xylose	0	0.05	0.03	0.26	0.05	0.50	0.15	1.50	0.33	3.30	1.17	11.70	1.85	18.50	1.64	16.40
Glucose	0.19	1.92	0.14	1.40	0.12	1.20	0.19	1.90	0.15	1.50	0.30	3.00	0.20	2.00	0.23	2.30
Arabinose	0.54	5.41	0.51	5.10	0.57	5.70	0.68	6.80	0.73	7.30	0.39	3.90	0.31	3.10	0.16	1.60
Acetic acid	4.07	40.70	3.36	33.60	3.78	37.80	4.94	49.40	14.73	147.30	22.59	225.90	32.33	323.30	38.16	381.60
Furfural	0	0	0	0	0	0	0.88	8.80	2.47	24.70	7.30	73.00	14.13	141.30	22.53	225.30
5-HMF	0	0	0	0	0	0	0	0	0	0	0.37	3.70	0.60	6.00	1.00	10.00
Formic acid	8.06	80.64	8.07	80.70	8.12	81.20	8.40	84.00	11.66	116.60	13.27	132.70	17.06	170.60	18.03	180.30
Tannic acid	0.51	-	0.37	-	0.54	-	0.21	-	0.77	-	1.09	-	1.07	-	1.72	-

* The XOs was calculated by subtracting total xylose monomeric sugars obtained after hydrolyzing pretreatment liquid with 4% H_2_SO_4_ and monomeric sugars in the pretreatment liquid as suggested by Sluiter et al. [27].

**Table 3:** Effect of subcritical H_2_O-CO_2_ pretreated solids on cellulose crystallinity index, specific surface area and sugar yields.

Treatment Conditions	Untreated OPMF	Subcritical H_2_O	Subcritical H_2_O-CO_2_		
Temperature (°C)	-	150	150	170	190
Time (min)	-	60	180	40	60
Pressure (MPa)	-	0	5	3	3
Log, *R*o	-	3.25	3.73	3.66	4.43
CS_PCO2_	-	−1.16	−0.34	−0.61	0.06
pH	-	4.41	4.18	4.27	4.43
Solid recovery (%)	-	85.75	73.73	70.22	61.88
Cellulose (%)	23.58	22.61	28.29	29.24	36.67
Hemicellulose (%)	22.34	17.94	12.14	12.31	3.14
CrI (%)	52.35	62.35	58.92	59.10	63.47
SSA (m^2^ g^−1^)	2.33	8.17	17.11	8.18	20.22
Pore volume (cm^3^ g^−1^)	0.01	0.04	0.08	0.04	0.01
* Sugar yield (%)					
Glucose	15.60 ± 7.5	31.83 ± 3.9	68.72 ± 11.0	70.26 ± 4.4	84.65 ± 2.5
Xylose	5.65 ± 0.6	16.99 ± 3.2	28.05 ± 2.8	29.96 ± 0	5.43 ± 0.9

* Sugar yield obtained from the untreated sample of OPMF.

**Table 4:** Comparative analyses between subcritical H_2_O and subcritical H_2_O-CO_2_ pretreatments of OPMF on sugar yields.

Pretreatment/ References	This Study	[19]	This study	[19]
Temperature (°C)	150	150	170	180
Time (min)	180	180	40	20
Log, *R*o	3.73	3.73	3.66	3.66
Pressure (MPa)	5	-	3	-
Glucose yield (%)	68.7	50.0	70.3	61.0
